# Identification of age differences in cancer-related symptoms in women undergoing chemotherapy for breast cancer in China

**DOI:** 10.1186/s12905-023-02256-9

**Published:** 2023-03-10

**Authors:** Tingting Cai, Tingting Zhou, Jialin Chen, Qingmei Huang, Changrong Yuan, Fulei Wu

**Affiliations:** School of Nursing, Fudan University, 305 Fenglin Road, Shanghai, 200032 China

**Keywords:** Breast cancer, Symptom, PROMIS, Age, Latent class analysis, Network analysis

## Abstract

**Background:**

Both contextual and cancer-related factors could be identified as causes of the interindividual variability observed for symptoms experienced during breast cancer treatment with chemotherapy. Understanding age differences and the predictors of latent class memberships for symptom heterogeneity could contribute to personalized interventions. This study aimed to identify the role of age differences on cancer-related symptoms in women undergoing chemotherapy for breast cancer in China.

**Methods:**

A cross‑sectional survey was conducted among patients with breast cancer in three tertiary hospitals in central China between August 2020 to December 2021. The outcomes of this study included sociodemographic and clinical characteristics, Patient-Reported Outcomes Measurement Information System (PROMIS)-57 and PROMIS-cognitive function short form scores.

**Results:**

A total of 761 patients were included, with a mean age of 48.5 (SD = 11.8). Similar scores were observed across age groups for all symptoms except for fatigue and sleep disturbance domains. The most central symptoms varied among each group, and were fatigue, depression, and pain interference for the young-aged, middle-aged, and elderly-aged groups, respectively. In the young-aged group, patients without health insurance (OR = 0.30, *P* = 0.048) and in the fourth round of chemotherapy or above (OR = 0.33, *P* = 0.005) were more likely to belong to low symptom classes. In the middle-aged group, patients in menopause (OR = 3.58, *P* = 0.001) were more likely to belong to high symptom classes. In the elderly-aged group, patients with complications (OR = 7.40, *P* = 0.003) tended to belong to the high anxiety, depression, and pain interference classes.

**Conclusions:**

Findings from this study indicated that there is age-specific heterogeneity of symptoms present for Chinese women being treated for breast cancer with chemotherapy. Tailored intervention should consider the impact of age to reduce patients’ symptom burdens.

## Background

Patients on chemotherapy for breast cancer experience multiple concurrent and coexisting symptoms caused by the disease and treatment toxicity [1]. Symptoms can result in treatment delays and non-adherence, with negative impacts on survival [2]. Age differences in treatment-related symptom burden are not well-documented in patients with breast cancer. The best survival rates of breast cancer have been found in middle-aged women, with decreased survival at each end of the age spectrum [3]. Young women with breast cancer have aggressive features and worse prognosis compared to older patients [4]. Evidence has shown that young women with breast cancer have worse health outcomes in terms of physical and psychosocial aspects, and chemotherapy was a significant treatment factor associated with poorer health outcomes [3, 5]. Additionally, chemotherapy played a more critical role in comparison to surgery, with age having an influence on the effects [3]. Older women with breast cancer were less likely to be concerned about premature menopausal symptoms, infertility, sexual dysfunction, and had less psychological symptoms [6]. Identification of age differences and patients at highest risk is critical and would benefit these patients by allowing early intervention. The modifiable drivers of symptom burden inequities can be reduced by minimizing the present knowledge gap.

Interindividual variability exists in the symptom experience for patients with breast cancer receiving chemotherapy. Contextual and cancer-related factors could both play different roles in this variability [7]. No studies to date have examined the age difference on symptoms via a person-centered method. Latent class analysis (LCA) is a person-centered approach that has been extensively used for clinical purposes and contributes to distinguishing those patients at risk [8, 9]. Additionally, intervention on central symptoms in the networks can provide targeted strategies. Cancer-related symptoms, and contextual and illness-related factors in patients with breast cancer display complex reciprocal interactions, which add to heterogeneous presentations of symptoms. The network approach has been used to examine multiple, complex relationships between symptoms among patients with chronic diseases, and helps to identify the most relevant connections between symptoms [10, 11]. Therefore, a LCA method in addition to network analysis is ideal for identifying symptom heterogeneity and the most central symptoms for appropriate intervention. Using LCA and network analysis, this study aimed to extend previous findings by identifying age difference and unique associations between cancer-related symptoms in women undergoing chemotherapy for breast cancer.

## Methods

### Study design and participants

This cross-sectional study recruited patients from three tertiary grade A hospitals (Affiliated Cancer Hospital of Fudan University, Affiliated Zhongshan Hospital of Fudan University, The Second Affiliated Hospital of Guilin Medical University) from August 2020 to December 2021. We invited women aged 18 or older who had been diagnosed with breast cancer, currently undergoing chemotherapy, and had access to a mobile app to complete a web-based survey. Patients were excluded if they were unable to participate owing to psychiatric or intellectual disabilities.

Patients were classified into three age groups: young-aged group (18–39 years), middle-aged group (40–59 years), elderly-aged group (older than 60 years). The study was approved by the institutional review boards of Fudan University and all corresponding hospitals (no.: 1810192–22). All participants were informed of the study aims and procedures, and signed written informed consent before the study. A web-based survey was performed and collected from the participants. The research assistant clarified each question raised by participants and checked medical records for completeness and consistency.

### Measures

Demographic and clinical characteristics of the patients were recorded in this study. The Patient-Reported Outcomes Measurement Information System (PROMIS) instruments, PROMIS-57 and PROMIS-cognitive function short form were included, with permission from the PROMIS National Center, China. The surveys took approximately 20–30 min to complete.

#### Demographic and clinical characteristics

Demographic characteristics included age, marital status, number of children, menstrual status, educational attainment, occupation, annual family income, and medical insurance. Clinical information, retrieved from the medical records, included complication status, therapeutic regimen, and chemotherapy cycles.

##### PROMIS-57

The PROMIS-57 was used to assess symptoms in this study. The scale consists of 57 items clustered into seven domains: anxiety, depression, fatigue, sleep disturbance, pain interference and intensity, physical function, and ability to participate in social roles and activities [12]. Each item was scored on a five-point Likert scale, except for pain intensity with one item, which was scored between 0 and 10 (least to most severe) [13]. Raw scores varied from 8 to 40 in each domain and were derived as per the PROMIS scoring manual into T-scores with a mean of 50 and a standard deviation (SD) of 10. Higher scores indicated a higher level of functioning or greater symptom severity. An acceptable internal consistency for the scale was found in this sample (α ranged from 0.87 to 0.97).

#### PROMIS-cognitive function short form

The 4-item PROMIS-cognitive function short form was used to identify perceived cognitive difficulties in the previous 7 days [14]. Items were scored on a 5-point Likert scale ranging from 1 (“very often”) to 5 (“never”) [15]. Total scores varied from 4 to 20 and were subsequently converted into T-scores (mean = 50, SD = 10). A higher score indicated better perceived cognitive function [15]. The Cronbach's α was 0.96 in this study.

### Statistical analyses

Statistical analyses were performed by using SPSS Statistics for Windows, version 26.0 (IBM Corp., Armonk, NY, USA), R version 4.1.0 and Mplus version 8.0. Descriptive analysis was used for the distribution of sociodemographic, clinical, symptoms, and function characteristics. Categorical variables were presented as frequencies and percentages, and continuous variables as means and SDs. A symptom network analysis was used to identify the most central symptom in the entire sample and in each age group. In the symptom networks, a node indicates an independent symptom, an edge indicates the conditional relationships between two symptoms, and the edge thickness shows the strength of the relationship between them [16]. Thus, two centrality indices (strength and closeness) were output to quantify the relationship. The strength value represents the probability of one symptom and other symptoms occurring together, and the closeness value represents the path from one symptom to all other symptoms [16].

The questionnaires were scored according to the PROMIS Scoring Manual, and were dichotomized as 0 or 1 according to the cutoff scores for clinical differences (https://www.healthmeasures.net/). After data processing, LCA was performed to identify clusters of individuals displaying similar patterns of symptoms by age groups (15–39, 40–59, and over 60 years). Models with an increasing number of latent classes were assessed until the best fitting model was determined. To select the optimal LCA model, the following indices were included: the Akaike information criterion (AIC), Bayesian information criterion (BIC), and adjusted BIC (aBIC) were used to assess information criteria; and the Lo-Mendell-Rubin (LMR) test and bootstrapped likelihood ratio test (BLRT) were used to improve the model fit, with significant values indicating a better fit for the k-class model than the k-1-class model. Entropy values that exceed 0.80 indicate a satisfactory classification accuracy [17]. Among the LCA models with different numbers of latent classes, a lower AIC, BIC, aBIC, larger entropy, and significant LMR-LRT and BLRT *p* values were indicative of good model fit [18]. Clinical interpretability was also considered to decide the best option. After the optimal model was determined, between-group difference was examined using Chi-square tests, Fisher’s exact tests or analysis of variance (ANOVA) where appropriate. Only statistically significant variables were entered into the stepwise logistic regression model. The regression was conducted separately by age groups to determine the contributing factors of symptoms for each group. *P* < 0.05 was considered statistically significant.

## Results

### Characteristics of the sample

Among the 803 participants investigated, 42 of them were not involved in the final analysis due to too many missing items. A final sample of 761 patients was included, with a mean age of 48.5 (SD = 11.8). Most of the patients were married, had children, were premenopausal, had a secondary education, were unemployed, had an annual family income of less than ¥60,000, had employment health insurance, were without complications, and had received chemotherapy combined with surgery. Among the 761 included patients, 217 (28.5%) belonged to the young-aged group (mean = 34.3, SD = 3.6), 397 (52.2%) belonged to the middle-aged group (mean = 50.0, SD = 5.6), and 147 (19.3%) belonged to the elderly-aged group (mean = 65.5, SD = 4.9). The detailed demographic and clinical characteristics of the participants are reported in Table 1.

**Table 1:** Demographic and clinical characteristics in the entire sample and by age groups

Characteristics	Entire sample (*n* = 761)	Young-aged group (*n* = 217)	Middle-aged group (*n* = 397)	Elderly-aged group (*n* = 147)	*P* value
**Age (mean ± SD)**	48.5 ± 11.8	34.3 ± 3.6	50.0 ± 5.6	65.5 ± 4.9	
**Marital status**					< 0.001
Single	23 (3.0%)	22 (10.1%)	1 (0.3%)	0	
Married	692 (91.0%)	188 (86.8%)	375 (94.4%)	129 (87.8%)	
Divorced	26 (3.4%)	7 (3.1%)	15 (3.8%)	4 (2.7%)	
Widowed	20 (2.6%)	0	6 (1.5%)	14 (9.5%)	
**Number of children**					< 0.001
No	45 (5.9%)	36 (16.6%)	7 (1.8%)	2 (1.4%)	
One child	392 (51.5%)	85 (39.2%)	245 (61.7%)	62 (42.2%)	
Two children	259 (34.1%)	81 (37.3%)	121 (30.5%)	57 (38.8%)	
Three or more children	65 (8.5%)	15 (6.9%)	24 (6.0%)	26 (17.6%)	
**Menstrual status**					< 0.001
Premenopausal	343 (45.1%)	192 (88.5%)	150 (37.8%)	1 (0.7%)	
Menopausal	104 (13.6%)	8 (3.7%)	67 (16.9%)	29 (19.7%)	
Postmenopausal	314 (41.3%)	17 (7.8%)	180 (45.3%)	117 (79.6%)	
**Educational attainment**					< 0.001
Primary school or below	147 (19.3%)	10 (4.6%)	84 (21.2%)	53 (36.1%)	
Secondary school	243 (31.9%)	60 (27.6%)	136 (34.2%)	47 (32.0%)	
High school	155 (20.4%)	36 (16.6%)	88 (22.2%)	31 (21.1%)	
University or above	216 (28.4%)	111 (51.2%)	89 (22.4%)	16 (10.8%)	
**Occupation**					< 0.001
Employed	145 (19.1%)	76 (35.0%)	66 (16.6%)	3 (2.0%)	
Medical leave	151 (19.8%)	59 (27.2%)	85 (21.4%)	7 (4.8%)	
Unemployed	274 (36.0%)	80 (36.9%)	152 (38.3%)	42 (28.6%)	
Retired	191 (25.1%)	2 (0.9%)	94 (23.7%)	95 (64.6%)	
**Annual family income (¥)**					< 0.001
< 60,000	455 (59.8%)	103 (47.5%)	243 (61.2%)	109 (74.1%)	
60,000–120,000	217 (28.5%)	80 (36.8%)	105 (26.5%)	32 (21.8%)	
> 120,000	89 (11.7%)	34 (15.7%)	49 (12.3%)	6 (4.1%)	
**Medical insurance**					0.001
Employed health insurance	285 (37.5%)	100 (46.1%)	139 (35.0%)	46 (31.3%)	
Urban health insurance	275 (36.1%)	69 (31.8%)	141 (35.5%)	65 (44.2%)	
Rural health insurance	160 (21.0%)	31 (14.3%)	96 (24.2%)	33 (22.5%)	
Without health insurance	41 (5.4%)	17 (7.8%)	21 (5.3%)	3 (2.0%)	
**Complication**					0.766
No	649 (85.3%)	180 (82.9%)	340 (85.6%)	129 (87.8%)	
Yes	112 (14.7%)	37 (17.1%)	57 (14.4%)	18 (12.2%)	
**Therapeutic regimen**					0.009
Chemotherapy + surgery	295 (38.8%)	74 (34.1%)	151 (38.0%)	70 (47.6%)	
Chemoradiotherapy + surgery	179 (23.5%)	45 (20.7%)	97 (24.4%)	37 (25.2%)	
Chemotherapy + surgery + endocrine therapy	198 (26.0%)	75 (34.6%)	97 (24.4%)	26 (17.7%)	
Chemotherapy + other	89 (11.7%)	23 (10.6%)	52 (13.2%)	14 (9.5%)	
**Chemotherapy cycles**					< 0.001
First cycle	220 (28.9%)	50 (23.0%)	118 (29.7%)	52 (35.4%)	
Second cycle	84 (11.0%)	31 (14.3%)	43 (10.8%)	10 (6.8%)	
Third cycle	62 (8.2%)	34 (15.7%)	21 (5.3%)	7 (4.7%)	
Fourth cycle or above	395 (51.9%)	102 (47.0%)	215 (54.2%)	78 (53.1%)	

### Symptoms among different age groups

As shown in Table 2, the average T scores of symptoms were all beyond the normal range according to the PROMIS score manual (mean = 50, SD = 10 for normal range). Significant differences were noted for the fatigue and sleep disturbance domains across the age groups (*P* < 0.05). Additionally, post hoc multiple comparisons demonstrated that the fatigue levels of the young and middle-aged groups were higher than those of the elderly-aged group, and the sleep disturbance scores of the middle-aged and elderly-aged groups were higher than those of the young-aged group.

**Table 2:** Symptom scores in the entire sample and by age groups

Symptom scores (mean ± SD)	Entire sample (*n* = 761)	Young-aged group (*n* = 217)	Middle-aged group (*n* = 397)	Elderly-aged group (*n* = 147)	*P* value
Anxiety	53.2 ± 10.6	54.1 ± 10.8	52.6 ± 10.0	53.6 ± 11.9	0.223
Depression	51.7 ± 9.2	51.8 ± 9.2	52.0 ± 9.1	50.8 ± 9.4	0.443
Fatigue	48.6 ± 7.6	48.3 ± 7.4	49.3 ± 7.4	47.3 ± 8.1	0.019
Sleep disturbance	50.3 ± 8.0	49.0 ± 8.0	50.6 ± 8.2	51.3 ± 7.6	0.017
Pain interference	52.7 ± 8.6	52.3 ± 8.4	53.2 ± 9.0	52.2 ± 8.1	0.336
Pain intensity	2.1 ± 1.9	2.2 ± 2.0	2.1 ± 1.8	1.8 ± 1.7	0.167

### Network analysis of symptoms across different age groups

**Fig. 1:**
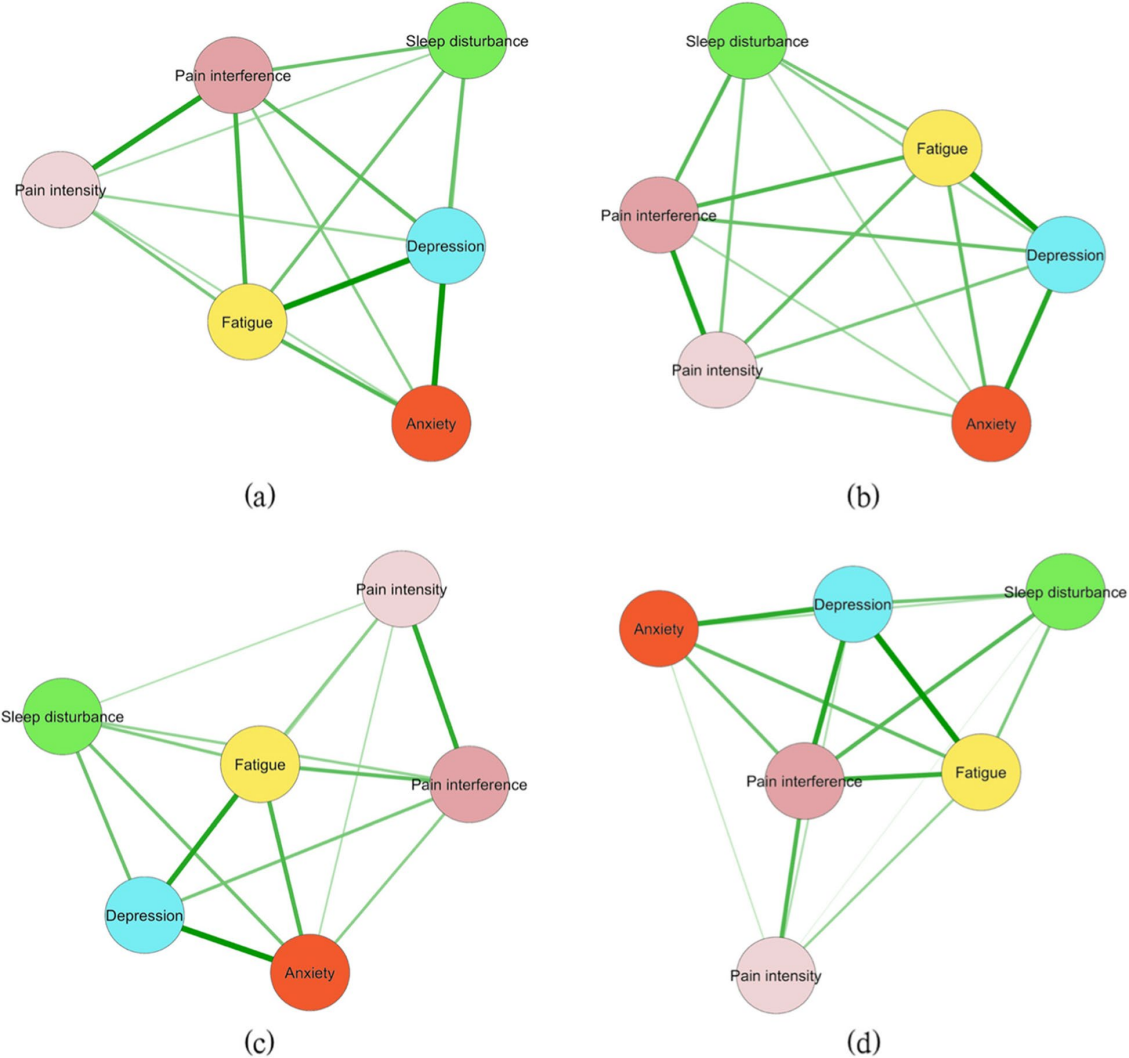
Symptom network analysis among the three age groups. **a** Entire sample; **b** Young-aged group; **c** Middle-aged group; **d** Elderly-aged group

Networks are depicted in Fig. 1 a-d, with each group across the entire sample represented. Between-group differences were identified in terms of symptom networks of varied populations. Among the entire sample, depression (*r*_s_ = 2.74, *r*_c_ = 0.10) was identified as the most central symptom according to two centrality indices, followed by fatigue (*r*_s_ = 2.65, *r*_c_ = 0.10). In the young-aged group, the most central symptom was fatigue (*r*_s_ = 2.55, *r*_c_ = 0.99), followed by depression (*r*_s_ = 2.52, *r*_c_ = 0.10). The most central symptom was depression (r_s_ = 2.92, r_c_ = 0.11), followed by fatigue (*r*_s_ = 2.80, *r*_c_ = 0.11) in the middle-aged group. In the elderly-age group, the most central symptom was pain interference (*r*_s_ = 2.72, *r*_c_ = 0.11), followed by depression (*r*_s_ = 2.70, *r*_c_ = 0.10).

### Latent class analysis among different age groups

**Fig. 2:**
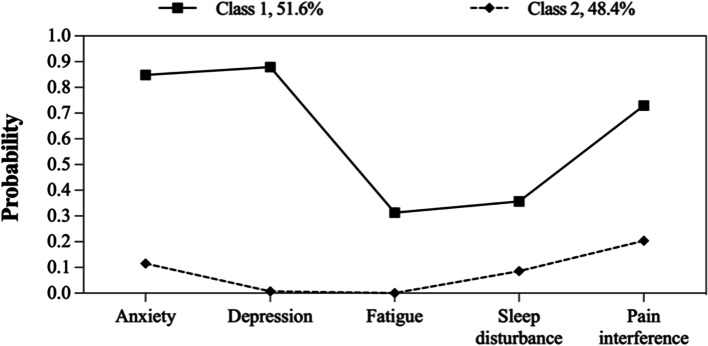
Item probabilities of latent classes in the young-aged group

**Fig. 3:**
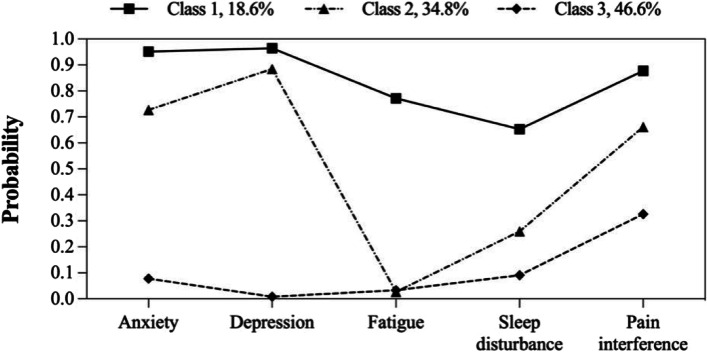
Item probabilities of latent classes in the middle-aged group

**Fig. 4:**
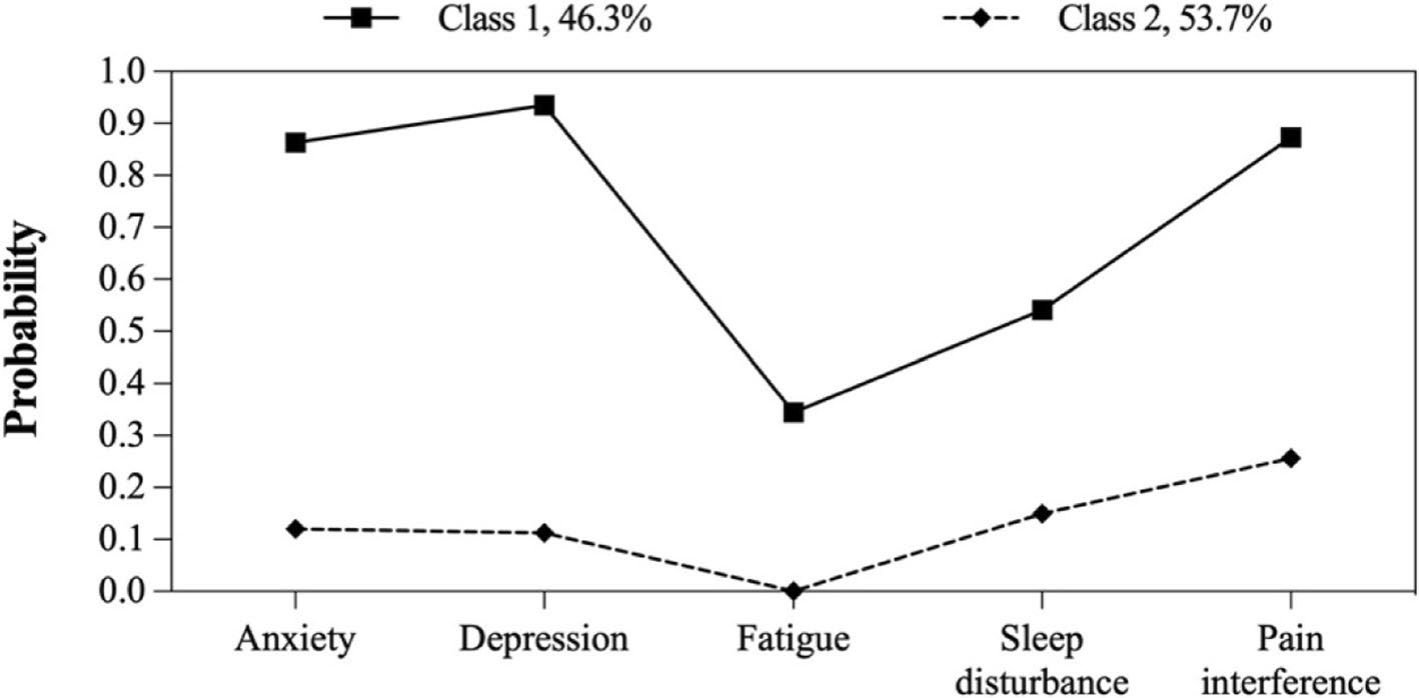
Item probabilities of latent classes in the elderly-aged group

The model fit statistics of LCA for each age group are reported in Table 3. The two-class model, the three-class model and the other two-class model were respectively selected by the young-aged group, the middle-aged group and the elderly-aged group, which was based on the model selection criteria for relatively low AIC, BIC, and aBIC values, high entropy, significant *P* values for LMR and BLRT, in addition to clinical interpretability. Detailed item probabilities of latent classes in different age groups were shown in Fig. 2, Fig. 3, and Fig. 4.

**Table 3:** Latent class model fit comparison

Model	Log	AIC	BIC	aBIC	Entropy	LMR *P* value	BLRT *P* value	Latent class probability
**Young-aged group**								
1C	-661.9	1333.8	1350.7	1334.8	-	-	-	1
2C	-552.4	1126.8	1164.0	1129.1	0.865	< 0.001	< 0.001	0.516/0.484
3C	-541.2	1116.3	1173.8	1119.9	0.753	0.068	< 0.001	0.359/0.207/0.434
4C	-538.1	1122.2	1199.9	1127.0	0.805	0.605	0.070	0.046/0.359/0.175/0.420
5C	-536.4	1130.9	1228.9	1137.0	0.836	0.060	0.082	0.166/0.046/0.175/0.419/0.194
**Middle-aged group**								
1C	-1258.2	2526.3	2546.2	2530.4	-	-	-	1
2C	-1044.0	2110.0	2153.8	2118.9	0.841	< 0.001	< 0.001	0.443/0.557
3C	-1030.8	2095.7	2163.4	2109.4	0.778	0.003	< 0.001	0.186/0.348/0.466
4C	-1028.0	2102.0	2193.6	2120.6	0.740	0.114	0.413	0.247/0.290/0.448/0.015
5C	-1026.5	2111.0	2226.5	2134.5	0.758	0.388	0.667	0.065/0.448/0.015/0.290/0.182
**Elderly-aged group**								
1C	-458.4	926.8	941.8	926.0	-	-	-	1
2C	-375.4	772.7	805.6	770.8	0.827	< 0.001	< 0.001	0.463/0.537
3C	-367.1	768.2	819.0	765.2	0.948	0.062	< 0.001	0.510/0.152/0.338
4C	-362.5	771.0	839.8	767.0	0.929	0.008	0.150	0.170/0.211/0.415/0.204
5C	-360.2	778.3	865.1	773.3	0.803	0.110	0.116	0.231/0.048/0.231/0.340/0.150

In terms of the latent classes in the young-aged group, Class 1 (*n* = 112, 51.6%) was characterized by high anxiety, depression, and pain intensity, as well as moderate fatigue and sleep disturbance, while Class 2 (*n* = 105, 48.4%) was characterized by low symptom probabilities. Similar patterns were found in the elderly-aged group (Class 1: *n* = 68, 46.3%; Class 2: *n* = 79, 53.7%). In the middle-aged group, an additional class with high symptom probabilities was identified: Class 1 (*n* = 74, 18.6%) was characterized by high symptom probabilities; Class 2 (*n* = 138, 34.8%) was characterized by high anxiety, depression and pain intensity but low fatigue and sleep disturbance, while Class 3 (*n* = 185, 46.6%) was characterized by low symptom probabilities.

### Predictors of the latent classes among different age groups

Univariate analysis was performed to identify between-group differences in the latent classes. The number of children, educational attainment, medical insurance, and chemotherapy cycles were identified as significant variables in the young-aged group, and menstrual status was identified in the middle-aged group. Additionally, the number of children and complication status were found to be statistically significant in the elderly-aged group.

Based on the significant variables by univariate analysis, logistic regression analysis was performed to identify potential predictors for higher risk classes, with the low symptom latent class of each age group set as the reference. The results are reported in Table 4. In the young-aged group, patients without health insurance (OR = 0.30, *P* = 0.048) and in the fourth chemotherapy or above (OR = 0.33, *P* = 0.005) were more likely to belong to low symptom classes. In the middle-aged group, menopausal patients (OR = 3.58, *P* = 0.001) were more likely to belong to high symptom classes. As for patients in the elderly-aged group, those with complications (OR = 7.40, *P* = 0.003) were more likely to report high anxiety, depression, and pain interference.

**Table 4:** Potential predictors of latent class membership among different age groups

**Young-aged group**			
**Characteristics**	**Class 1 (*n* = 112)**		***P* value**
	**OR**	**95% C.I**	
**Medical insurance**			
Employed health insurance	reference		
Urban health insurance	0.97	0.46–2.04	0.934
Rural health insurance	0.50	0.17–1.46	0.205
Without health insurance	0.30	0.09–0.99	0.048
**Chemotherapy cycles**			
First cycle	reference		
Secondary cycle	1.07	0.39–2.94	0.899
Third cycle	0.57	0.19–1.66	0.299
Fourth cycle or above	0.33	0.16–0.72	0.005
**Middle-aged group**			
**Characteristics**	**Class 1 (*n* = 74)**		***P* value**
	**OR**	**95% C.I**	
**Menstrual status**			
Premenopausal	reference		
Menopausal	3.58	1.69–7.59	0.001
Postmenopausal	1.11	0.58–2.10	0.761
**Elderly-aged group**			
**Characteristics**	**Class 1 (*n* = 68)**		***P* value**
	**OR**	**95% C.I**	
**Complication**			
No	reference		
Yes	7.40	1.98–27.60	0.003

## Discussion

This study examined whether age difference existed in terms of cancer-related symptoms in women with breast cancer receiving chemotherapy. Except for fatigue and sleep disturbance, similar symptom patterns were observed across the three age groups. Despite similar mean scores across the age groups, heterogeneity of symptoms was observed via the network analysis and the LCA. The results of network analysis demonstrated that depression and fatigue were the central symptoms in the investigated population. Depression and fatigue were frequently reported symptoms in patients with breast cancer, and younger women consistently had more severe symptoms when compared with their older counterparts [19, 20] In our study, pain interference was more closely related to the occurrence of other symptoms in the elderly-aged group. Furthermore, the results of the LCA also indicated that middle-aged patients showed more symptoms than other groups, in line with results from a prior study by Pinto et al. [21] showing that younger age (< 65 years) had a negative impact on physical and mental symptom domains.

Occupation, annual family income, and medical insurance were associated with symptoms in the investigated population. Employment was shown to play a significant role in post-diagnostic health according to existing evidence in patients with breast cancer [22]. Health benefits from employment included an increased sense of purpose, high self-esteem, and a strong sense of social support, all of which were associated with improved quality of life [23]. As a result of cancer and its expensive treatment, a financial dilemma was triggered owing to lower income, insufficient health insurance, or unemployment [24]. These results were consistent with previous reports in the context of cancer. For example, perceived financial hardship had been reported to be directly correlated with symptom distress in patients with advanced cancer [25]. In a sample of patients with breast cancer, the financial strain showed a significant association with worse depression, anxiety, and physical symptoms [26]. Further, a systematic review indicated that there was a positive relationship between financial strain and psychological symptoms including anxiety, depression, and overall distress, but less evidence for physical symptoms [27].

Young patients in the fourth chemotherapy cycle or higher appeared to belong to low symptom classes. Undergoing more chemotherapy cycles would allow the patients to be better prepared, and the findings were in accord with a previous study reporting that patients with breast cancer have more intense symptoms in the initial stage of chemotherapy [28]. However, young patients in this study did not report a higher symptom burden when compared with other groups. According to a systematic review, younger patients with breast cancer were at particular risk for psychological symptoms, especially for altered body image and sexual concerns, and the younger life stage was often associated with more aggressive treatments [29]. This inconformity might be attributed to the fact that one-tenth of the young patients were single; whereas in other groups there was a very small proportion of single patients.

For middle-aged patients, menopausal patients were more likely to report high symptom burdens. Menopausal symptoms such as fatigue, sleep disturbance, and anxiety, were common in patients after their chemotherapy and might be more frequent and severe among women after natural menopause [30]. Middle-aged patients were the groups with the largest proportion of low-income and rural health insurance in this study. Young to middle-aged patients were reported to experience more psychological stress than older counterparts, in part due to the effect of treatment on fertility, raising young children, not expecting to get a diagnosis of cancer at a relatively young age, worse marital satisfaction, and employment worries [31, 32].

Most notably, the presence of complications including myelosuppression, peripheral neurotoxicity, dermal toxicity, and urinary system toxicity significantly increased patients’ likelihood of reporting high anxiety, depression, and pain interference, particularly in the elderly-aged group. These women were more likely to have complications due to pre-existing medical conditions, which might cause psychological symptoms. The results were consistent with previous studies, showing that comorbidities and functional limitations were significant risk factors for depressive symptoms in older patients with breast cancer [27, 33]. Cancer and comorbidities have been reported to interact synergistically to affect physical and psychological outcomes in older patients with cancer [27, 33]. Having two or more comorbidities and functional limitations were strongly related to an elevated risk of depression both in patients with cancer and non-cancer controls, which supported our findings.

### Limitations

Several limitations must be noted in this study. First, the cross-sectional design might hinder our ability to explore the age differences in the symptom experiences over time. Second, the sample size was small, and the patients were recruited in tertiary grade A hospitals. The findings might not be generalizable to other care centers since results obtained by the network analysis and the LCA can be sample-specific. Finally, we did not specify the therapeutic regimen and chemotherapy drugs used in this study, which might have impacted the results. Further studies are needed to confirm the results in other populations.

## Conclusions

Findings from this study demonstrated symptom heterogeneity in women undergoing chemotherapy for breast cancer. Middle-aged patients, especially menopausal women, were more likely to report high symptom burdens. Additionally, older patients with complications were more likely to belong to have high levels of anxiety, depression, and pain interference classes. Tailored intervention should consider the impact of age to reduce symptom burdens.

## Data Availability

The dataset generated and/or analyzed during the current study are available from the corresponding author on a reasonable request.

## Abbreviations

PROMIS: Patient-reported outcomes measurement information system
LCA: Latent class analysis
AIC: Akaike information criterion
BIC: Bayesian information criterion
aBIC: Adjusted Bayesian information criterion
LMR test: Lo-Mendell-Rubin
BLRT: Bootstrapped likelihood ratio test
